# Reference genes for gene expression studies in the mouse heart

**DOI:** 10.1038/s41598-017-00043-9

**Published:** 2017-02-02

**Authors:** Adrián Ruiz-Villalba, Andrea Mattiotti, Quinn D. Gunst, Sara Cano-Ballesteros, Maurice J. B. van den Hoff, Jan M. Ruijter

**Affiliations:** 1 Department of Anatomy, Embryology and Physiology, Academic Medical Center, Amsterdam, The Netherlands; 2 Cell Therapy, Foundation of Applied Medical Research (FIMA), University of Navarra, Pamplona, Spain; 3 Department of Animal Biology, University of Málaga, Málaga, Spain

## Abstract

To be accurate, quantitative Polymerase Chain Reaction (qPCR) studies require a set of stable reference genes for normalization. This is especially critical in cardiac research because of the diversity of the clinical and experimental conditions in the field. We analyzed the stability of previously described as potential reference genes in different subsets of cardiac tissues, each representing a different field in cardiac research. The qPCR dataset was based on 119 different tissue samples derived from cardiac development to pathology in mouse adult hearts. These samples were grouped into 47 tissue types. The stability of 9 candidate genes was analyzed in each of 12 experimental conditions comprising different groupings of these tissue types. Expression stability was determined with the geNorm module of *qbase*+. This analysis showed that different sets of two or three reference genes are required for analysis of qPCR data in different experimental conditions in murine cardiac research.

## Introduction

Reverse transcriptase quantitative Polymerase Chain Reaction (qPCR) enables the quantification of differences in gene expression in tissues in a fast, sensitive and highly reproducible manner^1^. Therefore, qPCR assays are commonly used for gene expression studies and validation of high throughput transcriptome analyses. However, clinical and biological samples contain different tissues and cell types, each with unknown contribution to the observed gene expression. Moreover, each step of the tissue sampling, RNA isolation and reverse transcription process adds variation that is reflected in the amount of cDNA that is generated in the individual samples^2^. Therefore, the most important step in the analysis, from the viewpoint of accuracy, is a normalization strategy to correct for these unavoidable differences in sample size, sample composition and technical variations. This normalization should be done using a set of reference genes because no single gene can be assumed to be constant when experimental conditions affect tissue composition and gene expression levels simultaneously; by definition, a set of reference genes has an average expression that is independent of the biological process, treatment or disease that is studied^3^. The reliable identification of changes in expression levels of target genes depends on the stability of the reference genes and erroneous conclusions can be reached when the expression level of a target gene is determined with single and/or non-validated reference genes^4^.

This is especially relevant in cardiovascular research, where tissue samples that come from different developmental, pathological and experimental conditions are compared. During heart development a linear tube transforms into an adult four-chambered heart comprising changing heterogeneous cell populations^5^. This complexity further increases when pathological conditions and experimental models are included in the studies. Therefore, the need for validated stable reference genes in cardiac research is emphasized in literature but is generally not evaluated. As such, normalization remains an unsolved issue in many studies and remains a source of variation between studies conducted in different laboratories^6^.

In experimental research using mice, 18S rRNA, glyceraldehyde-3-phosphate dehydrogenase (Gapdh) or beta-actin (Actb)^7^ are most frequently used as single reference genes. However, several reports demonstrate that this choice leads to inadequate normalization. 18S rRNA, which amounts to almost halve of the total RNA in a cell, does not reliably reflect the changes in the mRNA pool that only contributes 1–2% to the RNA^3^. Gapdh encodes a glycolytic enzyme that is affected during heart failure^8^. Similarly, the expression level of the cytoskeleton protein Actb changes during heart development and failure^9^. Moreover, the large number of pseudogenes in the mouse genome negatively affects the reliability of Gapdh and Actb as reference genes when the PCR primers are not selected properly and the sample is contaminated with genomic DNA^10^.

These issues indicate that the choice of reference genes should not just be based on the similarity of the experimental design to earlier studies; reference gene stability should be determined in an experiment in which the stability of a set of candidate genes is tested in the full range of experimental conditions that are applied in the current study^3^.

In this paper, we determined the expression stability of 9 reference genes in a large set of tissue types, grouped into several experimental conditions that are frequently studied in cardiac research. These conditions range from embryonic development till the pathologic adult mouse heart. For each experimental condition the most stable combination of reference genes is reported.

## Results

### Stability analysis for all tissue samples

Melting curve analysis and agarose gel electrophoresis showed that all primer pairs led to amplification of the desired product in the different tissue samples provided that the samples were treated with DNAse. This DNAse treatment was essential to prevent amplification of genomic DNA (Supplemental Figure 1; after this analysis Actb was discarded). The median C_q_ values for the different candidate genes ranged from 19 to 27 whereas the median C_q_ difference between replicate tissue samples within a tissue type was between 0.1 and 0.8 cycles (Supplemental Table 1).

For each of the experimental conditions (Table 1; Supplemental Table 2) the reference gene stability was analyzed and the number of required reference genes was determined according to the default criteria of the geNorm module in *qbase*+ ^4^. The resulting M-values (indicating gene stability) and V-values (indicating the required number of reference genes) are summarized in Supplemental Figure 2. A summary of tissue types included in the experimental conditions, the stability values per gene and the minimal required number of genes is given in Table 1.

**Table 1:**
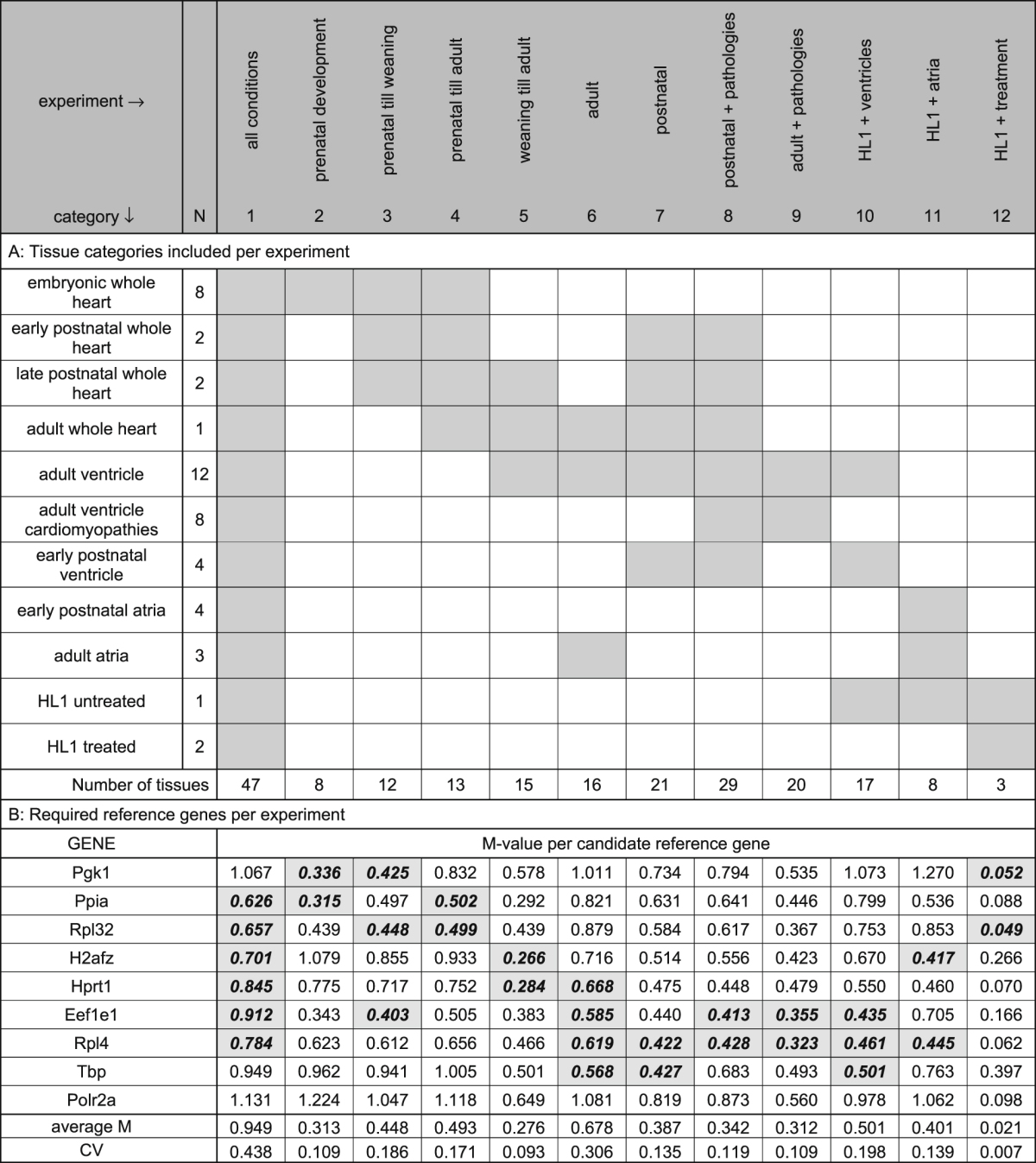
**A**. Grouping of categories of tissue types (rows) into experimental conditions (columns). Per category the number of included tissue types (N) are given. **B**. The reference genes required for normalization per experimental condition are indicated in gray; the M-values indicate the stability of the individual candidate reference genes in the experimental conditions. The average M-value and coefficient of variation (CV) are given for the required reference genes per experiment. See Supplemental Table 1 for an overview of the tissue samples included in each category of tissue types.

When all samples were analyzed as one set, none of the candidate genes reached an M-value below 0.5 although 7 genes had an M-value below 1.0 indicative of medium gene stability in a heterogeneous set of tissues^4^. The heart is indeed an organ which comprises many different cell types of which the relative contribution changes during development and disease. The combination of 6 genes reached a V-value below 0.15; this set of genes can serve to normalize expression levels in such an extensive collection of different cardiac tissue samples and pathologies (Table 1, column 1 ‘all conditions’). Because most experimental settings do not cover such an extensive variation of tissue types, the stability of the candidate reference genes was evaluated by grouping tissue types according to experimental conditions. In each of these groups, the number of samples fulfilled the criteria for geNorm analysis^4^.

### Stability analysis per experimental condition

The analysis of the different conditions showed that for each condition a different set of reference genes was required. When the samples of prenatal development (Table 1, column 2) were analyzed, two reference genes were found to be sufficient for normalization. However, when this prenatal group was extended to include samples until weaning (Table 1, column 3) or the adult stage (Table 1, column 4), a different set of two reference genes was found to be required. When the entire adult heart was grouped with samples only containing adult atria or ventricles the variability strongly increased, revealing the necessity of four reference genes (Table 1, column 6). The source of the variation was found to be the result of the addition of the atrial samples (data not shown). When the entire heart or ventricle samples of adult mice were grouped with those of late and early prenatal hearts (Table 1, column 5, 7) or cardiac pathologies (Table 1, column 8) the number of required references genes was found to be two. It should be noted that these reference genes are again different from the references genes required in the developmental groups (Table 1, columns 2–5), and are not always among the reference genes required for all conditions (Table 1, column 1). Interestingly, the analysis of the group “postnatal + pathologies” (Table 1, column 8) versus the group “adult pathologies” (Table 1, column 9) identified the same two reference genes. This result cannot be attributed to a biasing effect of the large number of adult tissue types included in these analyses because the adult whole heart category is included in four other experimental conditions which require different reference genes (Table 1, columns 5–7, 9, 10).

Samples obtained from cell lines can be considered more stable. Indeed, grouping the samples derived from treated and untreated HL1 cells showed high stability and required two reference genes (Table 1, column 12). The HL1 cell line is derived from adult mouse atrial cardiomyocytes^11^. When untreated HL1 cells are grouped with only adult atrial samples (Table 1, column 11) a higher stability of the potential reference genes is found than when grouped with adult ventricular samples (Table 1, column 10); the group “HL1 + ventricles” required three and the group “HL1 + atria” only two references genes for reliable normalization. These observations may reflect the atrial origin of the HL1 cells.

The analysis of this continuous series of tissue type combinations showed that the choice of the appropriate set of reference genes seems to be dependent on developmental and morphological clues. The restricted nature of the set of candidate genes precludes a full generalization. But nonetheless, some genes were often required when adult tissues were included (Tbp and Rpl4) whereas others were generally more stable in developmental series (Pgk1, Ppia and Rpl32) or pathological conditions (Eef1e1). When all tissue types are combined, all of these genes were required to form a stable normalization factor.

### Application of identified reference genes

After the identification of stable reference genes, two independent qPCR runs were performed in order to compare the expression level of the target gene Follistatin-like 1 (Fstl1) when normalized with the validated reference genes and the classically used reference gene Gapdh. In the first qPCR run hearts from embryonic day (E) 10.5 till adult (cf Table 1, column 4) and in the second qPCR run adult hearts with and without an induced myocardial infarction (cf Table 1, column 9) were measured. A one-way ANOVA test was performed to analyze the resulting gene expression data.

**Figure 1:**
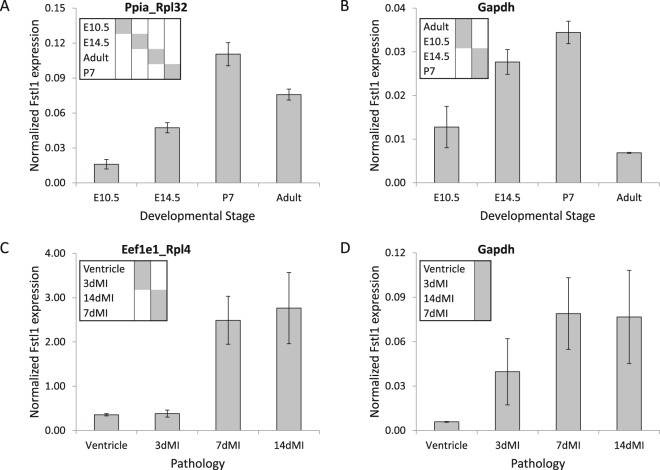
Effects of different normalization strategies on observed Fstl1 expression. Fstl1 expression in whole heart during development (embryonic day 10.5 and 14.5, postnatal day 7 and adult mouse) was determined with qPCR using Ppia and Rpl32 (**A**) or Gapdh as reference genes for normalization (**B**). Fstl1 expression in the normal left ventricle and at different days after induced myocardial infarction (MI) using Eef1e1 and Rpl4 (**C**) or Gapdh (**D**) as reference genes for normalization. The normalized Follistatin-like 1 expression differs significantly between groups (one-way ANOVA). The insets show the result of the subsequent multiple comparison between groups. Grey boxes indicate subsets of groups with absence of evidence for differential expression (p-value < 0.05) according the Student-Newman-Keuls test.

In the developmental series, the expression level of Fstl1 was found to differ significantly between the different developmental stages of the heart independent of the normalization approach (F = 41.439, p < 0.001, for Ppia and Rpl32 and F = 17.781, p < 0.001, for Gapdh). However, the expression pattern depended on the normalization strategy as is reflected by the post-hoc Student-Newman-Keuls test (p < 0.05, Fig. 1A,B). After normalizing with Ppia and Rpl32, the Fstl1 expression differed significantly between all studied stages (Fig. 1A). Using only Gapdh to normalize, the stages were divided in two subsets: the earliest and latest stages (E10.5 and adult) did not differ but were different from the intermediate stages (Fig. 1B).

In the myocardial infarction (MI) model a significant increase in Fstl1 expression at one and two weeks after MI was found when normalizing the data with the validated reference genes Eef1e1 and Rpl4 (F = 6.348, p = 0.011, Fig. 1C). These results are in accordance with those reported previously^12^. Using Gapdh as reference gene, a similar trend was observed but there was no significant difference between the four groups, due to the large within-group variation (F = 1.811, p = 0.209, Fig. 1D).

**Figure 2:**
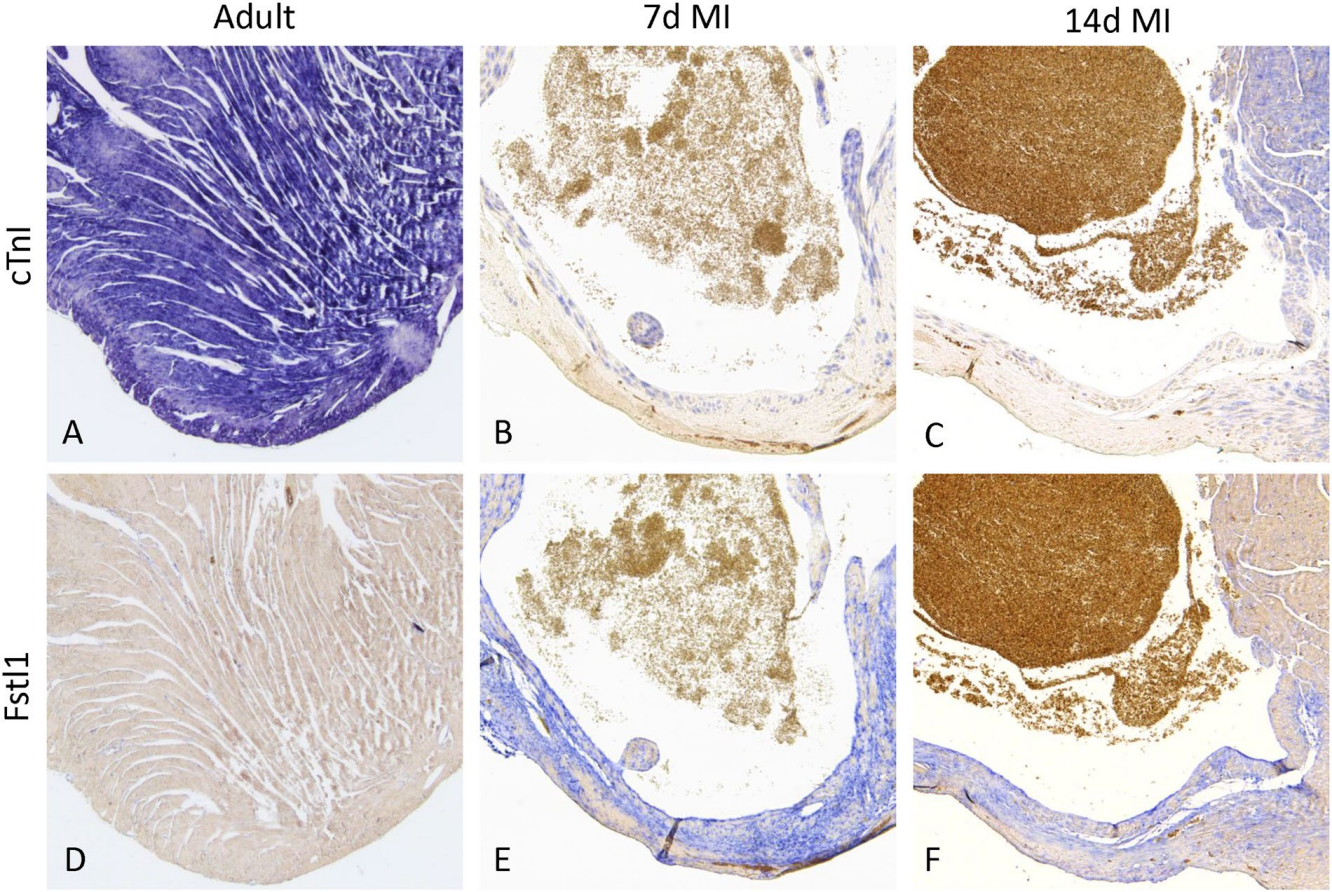
Fstl1 expression in the mouse heart after myocardial infarction (MI). *In situ* hybridization on sections was performed to visualize the expression of cTnI and Fstl1 in control ventricle (**A** and **D**) and after induced myocardial infarction (**B**,**E**,**C** and **F**). The infarcted area is identified by the absence of cTnI expression (**B** and **C**). Fstl1 expression is low in control heart (**D**) and highly expressed in the infarcted area at 7 days (**E**) and 14 days (**F**) after myocardial infarction (7d MI and 14d MI).

The results of the qPCR on Fstl1 were compared to *in situ* hybridization (ISH) on healthy and infarcted adult hearts. In the heart all cardiac muscle cells were visualized using a probe directed against cardiac Troponin I (cTnI), a component of the force generating sarcomeric complex (Fig. 2A). Upon induction a myocardial infarction (MI) in adult mice the ischemic myocardium almost instantaneously loses the expression of cTnI, which is retained in the remaining healthy myocardium (Fig. 2B,C); the non-stained region identifies the infarcted region of the ventricle. Fstl1 is expressed at low levels in the non-myocardial component of the heart (Fig. 2D). After induction of an MI the non-myocardial cells in the heart and especially those in the infarcted region start to express the Fstl1 (Fig. 2E,F). The ISH images confirm the high expression of Fstl1 shown at 7 and 14 days after the induction of the infarction with qPCR (Fig. 1).

Applying the ‘development’ reference genes to the pathology model, and vice versa, led to loss of most of the differences between groups; with the wrong set of reference genes, in both models only 1 group deviated from the other 3 (Supplemental Figure 3A,B).

## Discussion

In molecular biology the method of choice to determine gene expression levels is RT-qPCR. As in any other quantitative analysis an internal reference or standard is a prerequisite for accurate quantification. In RT-qPCR this internal reference is provided by the inclusion of one or several reference genes, which in older literature were erroneously dubbed house-keeping genes. The importance of the choice of stable reference genes for qPCR experiments cannot be underestimated^13^. This is especially relevant in cardiac research. With development, the heart traverses from a biosynthetic phase fashioned for hyperplastic growth, toward a mature phase in which the heart is optimally constructed for force production. Moreover, during development different extra-cardiac cells are found to invade the heart, resulting in changing relative contributions of the different cell types present within the heart. During disease the heart aims at preserving function by not only adapting gene expression but also by adapting the contribution of specific cell types. As a consequence differential regulation of the expression of reference genes is a potential pitfall in cardiac research. The changing contributions of different cell types in different cardiac compartments during developmental stages and pathologies make that either a large set of reference genes is required or that different sets of at least two reference genes are required for different experimental and clinical set-ups.^6,14^.

Several papers report on reference genes for heart infarction and hypertrophy models^6,14,15^. However, these studies were all restricted to two tissue types; tissue from sham operated mice compared to a single stage after treatment. The inclusion of such a small range of conditions may explain why the findings do not correspond to our current results. The data sets used for the identification of the required reference gene should include a full range of samples to represent the complete set of tissue types that is being compared in the final experiment^4^.

In this study, we used 119 different cardiac samples from 47 tissue types to evaluate the stability of 9 potential reference genes in 12 experimental conditions. To the best of our knowledge such a comprehensive analysis has not been reported, although the cardiac research field would benefit from standardized sets of reference genes for the evaluation of qPCR experiments. The inclusion of such a large set of tissue samples required the combination of data from different qPCR runs^16^. The applied removal of variation between samples per tissue type, which is the removal of variation introduced by the RT reaction, guarantees the minimization of variation between biological replicates and thus increases the power to detect differences between tissue types. This enables a more powerful analysis of gene stability.

In most experimental conditions only a few genes fulfil the strict stability criterion of an M-value below 0.5 although for most genes the M-value is below 1.0 as expected for heterogeneous sample sets^4^. Therefore, in some experimental conditions more than two reference genes are required to provide a stable normalization factor, sometimes including less stable reference genes which counteract each other’s effect. In order to be able to normalize gene expression levels of target genes in the entire data set, a group of six reference genes is needed. However, in case more restricted experimental conditions are analyzed it is possible to do experiments in which the expression levels of target genes can be normalized using two or three reference genes. Two points should be noted with respect to those reference genes. The combinations of two references genes required for reliable normalization are very specific for the combined groups of tissue samples; adding a single developmental stage, cardiac compartment or pathological condition to the set-up may require different reference genes. This observation also indicates that when experimental conditions that are not included in this study are used, such as specific transgenic models and other experimental treatments, the stability of these candidate genes needs to be re-confirmed.

The comparison of Fstl1 expression levels after normalization with classically used Gapdh on the one hand and the pairs of identified reference genes on the other, shows that only the use of the correct reference genes provides the sensitivity that enabled the distinction of the known developmental and pathological responses in the expression levels of Fstl1^12^. Normalization with Gapdh does not allow that level of precision. When the validated reference genes are used to normalize the expression Fstl1, the expression level found by qPCR is in line with the expression shown by ISH. However, when the non-validated Gapdh gene is used to normalize Fstl1, the qPCR data suggest that at 2 weeks post MI the expression of Fstl1 has already returned to control level, which is clearly not true considering the ISH result. This example shows that ISH, although this technique is not quantitative, can serve to confirm the results of a qPCR experiment. Indirectly such a confirmation also serves as an independent validation of the identified reference genes.

This study is about reference genes for qPCR. A qPCR experiment starts with a heterogeneous biological sample. Like all biochemical approaches, the resulting gene expression levels reflect the total sample, not the specific cellular components. Differential effects of the experimental treatment on the gene expression in different cell types in the tissue, or on their contribution to the tissue sample cannot be determined with qPCR analysis of the intact tissue. To address these questions tissue fractionation and cell sorting have to be performed before the RNA isolation and the qPCR analysis.

qPCR assays are commonly used for gene expression studies and validation of high throughput transcriptome analyses in cardiac research. The current study shows that choice of reference genes depends strongly on the experimental conditions included in these studies. The common use of a classical reference gene for all types of experiments may explain the difficulty to reproduce the results of these studies. Therefore, the field would benefit greatly when the cardiac research community would agree on using the same set of reference genes for normalization of gene expression data in similar experimental conditions. After doing so, the results obtained within one laboratory could be more easily compared with other laboratories. The use of common validated reference genes would result in better reproducibility between comparable studies in different laboratories.

## Methods

### Selection of reference genes

Potential reference genes were selected based on literature^6,14,15^. Nine candidate genes were selected to match the requirement for the analysis of reference gene stability^4^. Moreover, primers were obtained for the two classically used references genes, Gapdh and Actb, and a gene of interest, Fstl1. For each gene a pair of primers was designed using PrimerBlast (Genbank); primer information is summarized in Tables 2 and 3. Primers were tested on mouse cDNA, genomic DNA, a cDNA reaction without reverse transcriptase enzyme (-RT) and a non-tissue control (NTC). The specificity of qPCR reactions were analyzed using melting curve analysis and agarose gel electrophoresis.

**Table 2:** Gene information.

Gene name	Gene Symbol	Accession number NCBI	Length mRNA (bp)	Exons	Pseudo genes
Eukaryotic translation elongation factor 1 epsilon 1	Eef1e1	NM_025380.2	1039	4	None
Follistatin-like 1	Fstl1	NM_008047.5	3609	11	None
H2A histone family, member Z Isoforms 1 and 2	H2afz	NM_016750.3 NM_001316995.1	1069	5	2
Hypoxanthine guanine phosphoribosyl transferase	Hprt1	NM_013556.2	1349	9	2
Phosphoglycerate kinase 1	Pgk1	NM_008828.3	1840	11	11
Polymerase (RNA) II (DNA directed) polypeptide A	Polr2a	NM_001291068	6736	29	None
Peptidylprolyl isomerase A	Ppia	NM_008907.1	736	5	63
Ribosomal protein L32	Rpl32	NM_172086.2	504	4	7
Ribosomal protein L4	Rpl4	NM_024212.4	1422	10	3
TATA box binding protein	Tbp	NM_013684.3	1842	8	None
Glyceraldehyde-3-phosphate dehydrogenase isoform 1 and 2	Gapdh	NM_001289726.1 NM_008084.3	1296 1444	8	309
beta-Actin	Actb	NM_007393.5	1935	6	39

**Table 3:** PCR primers and amplicon information.

Gene Symbol	Sequences (5′to 3′)	Amplicon (bp)	Covering Exons	Location (nt)
Eef1e1	FW: TCCAGTAAAGAAGACACCCAGA	187	2–4	291–477
	RV: GACAAAACCAGCGAGACACA			
H2afz	FW: TAGGACAACCAGCCACGGA	217	3–5	324–540
	RV: GATGACACCACCACCAGCAA			
Fstl1	FW: AGCCCACGTGCCTCTGCATT	183	3–5	238–420
	RV: GGCTGGCAGATGGACTCGCA			
Hprt1*	FW: GCTTGCTGGTGAAAAGGACCTCTCGAAG	117	6–8	631–747
	RV: CCCTGAAGTACTCATTATAGTCAAGGGCAT			
Pgk1	FW: GTCGTGATGAGGGTGGACTT	126	1–3	206–331
	RV: AAGGACAACGGACTTGGCTC			
Polr2a	FW: CAACCAAGCCATTGCCCATC	135	19–20	3638–3772
	RV: ACACCCAGCGTCACATTCTT			
Ppia	FW: GGGTGGTGACTTTACACGCC	187	3–5	230–416
	RV: CTTGCCATCCAGCCATTCAG			
Rpl32	FW: GCCTCTGGTGAAGCCCAAG	177	2–3	66–242
	RV: TTGTTGCTCCCATAACCGATGT			
Rpl4	FW: GCCGCTGGTGGTTGAAGATAA	150	5–6	494–643
	RV: CGTCGGTTTCTCATTTTGCCC			
Tbp	FW: TATGACCCCTATCACTCCTG	250	3–6	613–862
	RV: TTCTTCACTCTTGGCTCCTGT			
Gapdh	FW: CTCCCACTCTTCCACCTTCG	189	6–7	1101–1289
	RV: GCCTCTCTTGCTCAGTGTCC			953–1141
Actb	FW: GGCTGTATTCCCCTCCATCG	154	2–3	193–346
	RV: CCAGTTGGTAACAATGCCATGT			

^*^Sequences obtained from ref. 18.

### Animals

All experimental procedures in this study were carried out conform the guide lines for the care and use of laboratory animals published by the US National Institutes of Health (NIH Publication No. 85-23, revised 1996) and the European Commission Directive 2010/63/EU. All experimental protocols were approved by the institutional animal ethics board of the Academic Medical Center, Amsterdam, NL. The approvals are registered under numbers DAE-100484, -000099 and -102701.

### Sample collection

Amplification data from a total of 119 different tissue samples from 47 different tissue types representing different prenatal and postnatal developmental stages (E10.5 to adult), cardiac regions and experimental conditions in mice, from two different genetic backgrounds (FVB and C57BL/6), were collected from a series of qPCR runs. Between 1 and 8 biological replicates of each tissue type were included (Supplemental Table 2).

### Quantitative Polymerase Chain Reaction (qPCR) and quality controls

Total RNA was isolated using TriReagent (Life Technologies) following the manufacturer’s protocol. For stages between E10.5 and E15.5 the tissue was homogenized using the Ultra-Turrax (IKA). For stages older than E16.5 the tissue was homogenized using the MagNA Lyser (Roche). RNA purity (A_260_/A_280_ ratio ≥ 1.8) and concentration were measured using the Nanodrop. Total RNA was stored at −80 °C. Prior to cDNA preparation, genomic DNA was removed using the Heat&Run gDNA removal kit (Articzymes). Between 300 and 1000 ng of total RNA was converted into cDNA using 125 μM of anchored oligo-dT primers (T_14_VT) and Superscript II reverse transcriptase (Life Technologies). Oligo-dT primer assured the selective transcription of the 3′ parts of mRNA; PCR primer pairs were designed accordingly. In each qPCR reaction the cDNA equivalent of 5 ng RNA was used. The qPCR reactions contained LightCycler 480 SYBR Green I Master (Roche) and the forward and reverse primer each at a final concentration of 0.25 or 1 μM. The amplification protocol consisted of 5 min 95 °C followed by 45 cycles of 10 sec 95 °C, 20 sec 60 °C and 20 sec 72 °C; and completed with a standard melting curve protocol. Melting curve analysis (LightCycler480, Roche) and size fractionation by agarose gel electrophoresis were used to confirm amplification of the expected product. The raw data were exported and analyzed using the *LinRegPCR* program^17^. Systematic differences induced by RT reactions in the observed expression levels per sample within tissue types as well as systematic differences between qPCR runs were removed using Factor-qPCR^16^. As all inter-run calibration methods, this program assumes a multiplicative between-run factor. To determine this factor for every run, the program uses all overlapping results between the different qPCR runs. It does not rely on specified samples but uses all data resulting from measurements of the same tissue type and gene that are thus replicated between the different PCR runs to construct a matrix of between-run ratios. The latter matrix then serves to calculate correction factors per run. In this between-run correction, the differences between tissue types and genes are preserved. After this correction, the samples per tissue type were considered technical replicates in the further analysis; the latter prevents bias by the variable number of biological samples per tissue. The dataset is available in RDML (Supplemental RDML file).

### *In situ* hybridization

*In situ* hybridization (ISH) was performed as described^12^. In short, the heart was isolated and fixed in freshly prepared 4% paraformaldehyde dissolved in phosphate buffered saline pH 7.6 (PBS). After embedding the tissue in paraplast, 10 μm thick sections were prepared. The sections were de-paraffinized and rehydrated. After Proteinase K treatment the sections were pre-hybridized in hybridization buffer (50% formamide, 20X SSC, blocking reagent, 0.5 M EDTA, 10% CHAPS, heparine solution and 10 mg/ml yeast RNA) at 70 °C. Subsequently, the sections were hybridized overnight with a dioxigenin (DIG) labeled probe directed against the cardiomyocyte specific cardiac Troponin I (cTnI) or Fstl1^12^. The next day, sections were extensively washed and the bound probe was immunohistochemically visualized using alkaline phosphatase-conjugated anti-DIG Fab fragments (Roche catalog number: 10932740) and NBT/BCIP staining reagent (Roche catalog number: 1681451). After staining the sections were dehydrated, mounted in Entellan and micrographs were acquired using a Leica DFC450 camera mounted on a Leica DM5000 microscope.

### Stability analysis

The C_q_ values per gene and sample, as well as the PCR efficiency per gene (Supplemental Table 3) were imported into the *qbase*+ program (Biogazelle)^4^. Using the geNorm module the most stable combination of reference genes for the total set as well as for subsets of samples reflecting specific experimental settings was determined (Supplemental Table 2). In short, the geNorm module calculates an M-value for each candidate reference gene using an algorithm that determines the geometric mean of its relative expression in each sample in a pairwise comparison with each other gene in the complete set of candidate genes. M-values below 0.5 are indicative of high reference genes stability which occurs in homogeneous sets of tissue types. In the case of heterogeneous tissues, like the heart samples used in this analysis, candidate genes with M-values up to 1.0 can be used^4^. A similar procedure serves to determine the stability of sets of genes based on the stepwise exclusion of the most unstable gene, resulting in a plot of the pairwise variation coefficients (V-values). The smallest set of genes with a V-value below 0.15 is considered a suitable set of reference genes to normalize gene expression in the given experimental setting. Note that such a set can include genes with an M-value of more than 0.5 which contribute to the stability of the set of reference genes.

## Electronic Supplementary Material

Supplementary Material
