# Supplementary material for: Reference genes for gene expression studies in the mouse heart
Source: Sci Rep. 2017 Feb 2;7:24. doi: 10.1038/s41598-017-00043-9 (PMC5428317; doi:10.1038/s41598-017-00043-9)

Reference genes for gene expression studies in the mouse heart

Adrián Ruiz-Villalba[1,2†], Andrea Mattiotti[1†], Quinn D Gunst[1], Sara Cano-Ballesteros[3], Maurice J van den Hoff[1], Jan M Ruijter[1*]

[1]Department of Anatomy, Embryology and Physiology, Academic Medical Center, Amsterdam, The Netherlands

[2]Cell Therapy, Foundation of Applied Medical Research (FIMA), University of Navarra, Pamplona, Spain

[3]Department of Animal Biology, University of Málaga, Málaga, Spain

[†]Authors contributed equally

Supplementary Material

Supplementary Table 1.
Minimum, mean and maximum observed Cq value for each of the candidate reference genes in each of the tissue types.

Gene→	Eef1e1			H2afz			Hprt1			Pgk1		
Tissue Type	Min	Mean	Max	Min	Mean	Max	Min	Mean	Max	Min	Mean	Max
14dBZ	24.8	25.3	26.2	21.9	22.5	23.0	22.4	22.8	23.5	22.3	22.6	22.8
14dIZ	29.7	29.7	29.7	24.6	24.6	24.6	28.3	28.3	28.3			
3dBZ	23.6	24.6	25.7	20.3	21.6	22.8	21.8	22.4	23.3	21.9	22.3	22.8
3dIZ	27.1	27.1	27.1	23.2	23.2	23.2	25.5	25.5	25.5	24.7	24.7	24.7
7dIZ	26.0	26.0	26.0	21.5	21.5	21.5	23.2	23.2	23.2	22.9	22.9	22.9
AdultLA	27.2	28.3	29.3	23.3	23.5	23.6	25.2	25.3	25.5	26.5	26.5	26.5
AdultLV	23.1	25.5	33.2	20.2	21.2	22.4	20.3	21.2	22.4	19.6	20.1	21.2
AdultRA	31.3	33.1	36.3	24.8	26.0	27.2	26.1	27.2	28.4	30.2	30.8	31.4
AdultWH	29.1	30.0	30.8	24.8	26.0	26.8	26.8	27.2	27.7	26.7	29.0	30.7
Atria	23.2	23.7	24.1				22.3	22.3	22.3	19.9	20.1	20.3
e10	25.6	26.3	26.9	20.9	20.9	20.9	24.4	25.0	25.6	22.2	22.6	23.1
e11	25.3	26.9	28.5	20.9	22.4	23.9	24.2	24.4	24.6	22.4	23.3	24.1
e12	23.7	24.1	24.5	19.1	19.1	19.1	22.5	23.0	23.5	20.8	20.9	21.0
e14	23.5	23.7	24.0	18.8	18.8	18.8	21.7	22.2	22.6	20.4	20.4	20.4
e15	35.7	35.7	35.7	31.5	31.5	31.5	32.5	33.1	33.7	31.6	32.0	32.4
e16	29.8	30.0	30.3	28.4	29.2	29.9	25.7	26.1	26.9	27.2	27.4	27.9
e17	29.0	29.0	29.0	23.6	23.6	23.6	25.4	25.4	25.4	22.8	22.8	22.8
e18	23.6	23.6	23.6	19.1	19.1	19.1	21.3	22.0	22.7	19.7	19.8	19.9
HL1	27.1	27.3	27.4	22.3	22.4	22.5	23.3	23.5	23.7	20.2	20.3	20.4
HL1Bmp	27.3	27.4	27.6	21.2	21.3	21.4	22.7	23.1	23.4	19.6	19.7	19.7
HL1Tsa	27.7	27.7	27.7	21.7	21.7	21.7	24.1	24.1	24.1	20.7	20.7	20.7
LVendoHETheart	24.2	24.8	25.4	21.5	21.7	21.9	21.8	22.2	22.6	19.9	20.9	21.8
LVendoWTheart	23.9	25.2	26.4	20.6	22.1	23.7	21.3	22.6	23.9	20.3	21.5	22.8
LVepiHETheart	24.2	24.3	24.4	21.2	21.2	21.2	21.7	21.8	21.8	20.7	20.9	21.1
LVepiWTheart	24.4	25.0	25.5	21.0	21.3	21.5	21.3	21.7	22.1	19.7	20.6	21.4
LVmiSham	23.4	24.0	24.5	20.9	21.4	21.9	21.5	21.7	21.9	20.5	20.5	20.6
LVtac	24.6	25.0	25.3	22.4	22.7	22.9	22.0	22.5	22.8	20.4	20.7	20.9
LVtrans	25.4	25.9	26.4	23.0	23.2	23.5	23.1	23.5	23.9	21.8	21.8	21.9
p14WH	25.4	25.7	26.1	22.0	22.0	22.0	22.4	22.8	23.2	21.8	22.0	22.2
P1LA	25.2	25.2	25.2	20.3	20.3	20.3	22.1	22.1	22.1	21.0	21.0	21.0
P1LV	29.1	29.1	29.1	23.5	23.5	23.5	26.2	26.2	26.2	26.9	26.9	26.9
P1RA	27.1	27.1	27.1	21.7	21.7	21.7	24.1	24.1	24.1	23.8	23.8	23.8
P1RV	27.9	27.9	27.9	22.3	22.3	22.3	23.2	23.2	23.2	21.7	21.7	21.7
p1WH	23.8	24.2	24.6	19.4	19.4	19.4	21.8	22.4	23.0	20.3	20.4	20.5
p21WH	24.3	24.7	25.0	21.5	21.5	21.5	21.7	22.3	22.9	20.6	20.9	21.2
P7LA	26.2	26.2	26.2	20.4	20.4	20.4	22.2	22.2	22.2	20.4	20.4	20.4
P7LV	32.8	33.1	33.4	27.9	29.0	30.0	29.2	30.1	31.1	31.6	31.9	32.2
P7RA	27.7	27.7	27.7	21.7	21.7	21.7	24.0	24.0	24.0	25.0	25.0	25.0
P7RV	26.6	26.6	26.6	21.4	21.4	21.4	23.1	23.1	23.1	21.3	21.3	21.3
p7WH	24.3	24.6	24.9	20.3	20.3	20.3	21.7	22.3	22.9	20.7	20.8	20.9
RVendoHETheart	24.1	24.6	25.1	20.9	21.4	21.9	21.1	21.7	22.3	20.0	20.9	21.7
RVendoWTheart	24.1	25.3	26.5	21.0	22.3	23.6	21.5	23.0	24.5	19.9	21.7	23.5
RVepiHETheart	24.2	24.7	25.2	21.1	21.7	22.4	21.7	22.1	22.6	20.5	20.8	21.0
RVepiWTheart	24.6	24.8	25.0	21.3	21.7	22.1	21.4	22.0	22.6	20.1	21.0	22.0
RVOTHET	23.8	24.1	24.5	20.7	21.3	22.0	21.3	22.0	22.6	19.6	20.9	22.2
RVOTWT	26.5	26.7	26.9	23.5	23.6	23.7	23.8	23.9	24.1	22.9	23.8	24.6
Ventricles	25.1	25.3	25.5	22.0	22.0	22.0	22.1	22.7	23.2	21.5	21.7	21.9

Supplementary Table 1 (continued).

Gene→	Polr2a			Ppia			Rpl32			Rpl4			Tbp		
Tissue Type	Min	Mean	Max	Min	Mean	Max	Min	Mean	Max	Min	Mean	Max	Min	Mean	Max
14dBZ	25.6	26.2	27.2	19.2	19.9	20.4	18.8	19.4	20.4	19.9	20.0	20.2	26.1	26.3	26.7
14dIZ	29.0	29.0	29.0	20.9	20.9	20.9	20.2	20.2	20.2	24.3	24.3	24.3	29.5	29.5	29.5
3dBZ	25.5	26.3	26.9	18.8	19.4	19.9	18.5	19.1	19.8	19.6	19.9	20.2	26.2	26.8	27.4
3dIZ	29.1	29.1	29.1	20.9	20.9	20.9	20.4	20.4	20.4	22.0	22.0	22.0	29.0	29.0	29.0
7dIZ	26.7	26.7	26.7	19.0	19.0	19.0	18.6	18.6	18.6	19.9	19.9	19.9	26.9	26.9	26.9
AdultLA	31.3	31.3	31.3	20.0	20.2	20.4	19.7	19.8	19.8	22.2	22.4	22.6	29.3	29.3	29.4
AdultLV	23.4	24.7	25.5	17.0	18.1	19.6	16.0	20.0	31.1	17.1	20.5	29.9	24.7	25.9	27.0
AdultRA	33.6	33.6	33.6	20.8	21.9	22.9	20.3	21.1	21.9	23.3	24.7	26.1	31.1	33.5	35.3
AdultWH	32.2	33.6	35.8	21.9	22.9	24.2	21.9	22.6	23.7	24.2	25.2	26.2	31.1	32.4	33.7
Atria	24.7	24.7	24.7	17.5	17.8	18.2	17.4	17.7	17.9	18.3	18.5	18.7	24.1	24.1	24.1
e10	28.2	28.2	28.2	19.1	19.6	20.2	19.4	19.5	19.7	20.5	20.9	21.2	27.6	27.6	27.6
e11	27.6	29.0	30.5	19.0	20.0	21.0	19.2	19.2	19.2	20.6	20.6	20.7	27.4	28.7	30.1
e12	26.1	26.1	26.1	17.3	17.6	17.9	17.6	17.8	18.1	18.9	19.1	19.3	25.6	25.6	25.6
e14	25.8	25.8	25.8	16.8	17.2	17.6	17.1	17.5	17.8	18.4	18.8	19.2	25.1	25.1	25.1
e15	30.7	32.8	35.0	28.2	28.3	28.4	29.0	29.2	29.4	32.4	32.4	32.5	34.5	34.5	34.5
e16	32.2	32.3	32.5	23.3	23.7	24.4	23.5	23.8	24.1	25.1	25.7	26.4	32.1	33.7	34.7
e17	27.3	27.3	27.3	20.9	20.9	20.9	20.5	20.5	20.5	21.9	21.9	21.9	28.8	28.8	28.8
e18	24.4	24.4	24.4	16.9	17.1	17.4	17.5	17.7	17.9	18.4	18.7	19.0	24.8	24.8	24.8
HL1	25.3	25.4	25.6	20.4	20.4	20.4	20.5	20.5	20.5	21.5	21.6	21.8	26.3	27.1	27.9
HL1Bmp	24.8	25.1	25.4	19.7	20.1	20.5	19.9	19.9	19.9	21.1	21.2	21.3	26.2	27.3	28.4
HL1Tsa	26.1	26.1	26.1	20.9	20.9	20.9	20.9	20.9	20.9	22.1	22.1	22.1	26.5	26.5	26.5
LVendoHETheart	24.4	24.6	24.8	18.5	19.1	19.7	18.5	18.8	19.2	18.8	19.3	19.8	25.2	25.3	25.3
LVendoWTheart	24.1	25.5	27.0	18.6	19.7	20.8	18.2	19.3	20.4	18.6	19.7	20.9	24.4	25.9	27.5
LVepiHETheart	25.1	25.2	25.2	18.9	19.0	19.2	18.0	18.5	19.0	18.5	19.0	19.5	24.9	25.1	25.2
LVepiWTheart	23.8	24.2	24.6	18.3	18.8	19.3	17.7	18.3	18.8	18.1	18.7	19.4	24.7	24.8	25.0
LVmiSham	23.7	23.8	23.8	18.9	18.9	19.0	18.3	18.5	18.7	18.7	18.7	18.7	24.8	25.0	25.2
LVtac	25.5	25.7	25.9	19.6	19.8	20.1	18.9	19.4	19.9	19.7	19.8	19.9	26.0	26.1	26.3
LVtrans	26.6	26.7	26.9	20.5	20.6	20.7	19.3	19.6	19.9	20.3	20.4	20.4	26.3	26.4	26.5
p14WH	26.8	26.8	26.8	19.7	19.9	20.2	19.2	19.4	19.6	19.9	20.2	20.5	26.3	26.3	26.3
P1LA	26.1	26.1	26.1	17.1	17.1	17.1	17.6	17.6	17.6	18.9	18.9	18.9	25.8	25.8	25.8
P1LV	31.2	31.2	31.2	21.0	21.0	21.0	21.5	21.5	21.5	22.8	22.8	22.8	30.9	30.9	30.9
P1RA	28.9	28.9	28.9	18.7	18.7	18.7	19.4	19.4	19.4	20.6	20.6	20.6	27.5	27.5	27.5
P1RV	26.4	26.4	26.4	18.7	18.7	18.7	19.1	19.1	19.1	20.7	20.7	20.7	27.0	27.0	27.0
p1WH	24.5	24.5	24.5	17.1	17.4	17.7	17.8	18.0	18.1	19.0	19.2	19.4	24.6	24.6	24.6
p21WH	25.2	25.2	25.2	19.3	19.5	19.6	18.7	18.8	18.9	19.3	19.5	19.8	25.3	25.3	25.3
P7LA	24.9	24.9	24.9	17.3	17.3	17.3	17.5	17.5	17.5	18.7	18.7	18.7	25.5	25.5	25.5
P7LV	36.4	36.4	36.4	24.1	24.7	25.4	24.2	24.3	24.4	26.6	27.3	27.9	32.9	34.0	35.1
P7RA	28.0	28.0	28.0	19.1	19.1	19.1	19.6	19.6	19.6	21.3	21.3	21.3	27.8	27.8	27.8
P7RV	25.1	25.1	25.1	18.5	18.5	18.5	19.2	19.2	19.2	19.5	19.5	19.5	26.6	26.6	26.6
p7WH	24.5	24.5	24.5	17.7	18.0	18.3	17.9	18.1	18.3	18.8	19.2	19.5	24.7	24.7	24.7
RVendoHETheart	24.7	25.1	25.5	18.5	19.2	19.8	18.2	18.8	19.4	18.3	19.1	20.0	24.7	25.2	25.8
RVendoWTheart	24.0	25.0	25.9	18.3	20.0	21.7	17.8	19.3	20.8	18.0	19.8	21.6	24.3	25.9	27.5
RVepiHETheart	24.3	25.5	26.7	19.0	19.3	19.7	18.6	18.6	18.6	19.3	19.3	19.4	24.9	25.1	25.3
RVepiWTheart	24.0	24.7	25.3	18.6	19.1	19.6	18.2	18.6	19.1	18.5	19.3	20.0	25.4	25.4	25.4
RVOTHET	24.8	25.0	25.2	18.0	18.9	19.9	17.9	18.5	19.2	18.3	19.1	20.0	24.7	25.2	25.7
RVOTWT	26.4	26.7	27.1	20.8	21.2	21.6	20.1	20.3	20.5	20.8	21.2	21.6	27.2	27.2	27.3
Ventricles	26.2	26.2	26.2	19.6	19.9	20.1	18.8	19.1	19.3	19.6	19.9	20.2	26.6	26.6	26.6

Supplementary Table 2

Overview of tissues samples per tissue type and category. For each category the included tissue types and number of tissue samples (N) per type are given. The number of qPCR reactions per tissue type (N) are specified per candidate reference gene. In the analysis, the reactions per gene and tissue type are treated as technical replicates.

category	tissue type	samples per type (N)	reactions (N)	reactions per gene								
				Eef1e1	H2afz	Hprt1	Pgk1	Polr2a	Ppia	Rpl32	Rpl4	Tbp
embyonic stages whole heart	e10.5	4	15	2	1	2	2	1	2	2	2	1
	e11.5	5	18	2	2	2	2	2	2	2	2	2
	e12.5	4	15	2	1	2	2	1	2	2	2	1
	e14.5	4	15	2	1	2	2	1	2	2	2	1
	e15.5	2	15	1	1	2	2	2	2	2	2	1
	e16.5	3	25	3	2	3	3	2	3	3	3	3
	e17.5	2	9	1	1	1	1	1	1	1	1	1
	e18.5	4	14	1	1	2	2	1	2	2	2	1
early postnatal whole heart	p1 WH	4	15	2	1	2	2	1	2	2	2	1
	p7 WH	4	15	2	1	2	2	1	2	2	2	1
early postnatal ventricles	P1 LV	1	9	1	1	1	1	1	1	1	1	1
	P1 RV	1	9	1	1	1	1	1	1	1	1	1
	P7 LV	1	19	2	2	3	2	1	2	2	2	3
	P7 RV	1	9	1	1	1	1	1	1	1	1	1
early postnatal atria	P1 LA	1	9	1	1	1	1	1	1	1	1	1
	P1 RA	2	9	1	1	1	1	1	1	1	1	1
	P7 LA	1	9	1	1	1	1	1	1	1	1	1
	P7 RA	1	9	1	1	1	1	1	1	1	1	1
late postnatal whole heart	p14 WH	4	15	2	1	2	2	1	2	2	2	1
	p21 WH	4	15	2	1	2	2	1	2	2	2	1
adult whole heart	Adult WH	5	44	5	4	5	5	5	5	5	5	5
adult ventricles	Ventricles	2	15	2	1	2	2	1	2	2	2	1
	Adult LV	2	39	5	4	4	4	4	4	5	5	4
	LV endo WT	1	18	2	2	2	2	2	2	2	2	2
	LV endo HET	1	18	2	2	2	2	2	2	2	2	2
	LV epi WT	1	18	2	2	2	2	2	2	2	2	2
	LV epi HET	1	18	2	2	2	2	2	2	2	2	2
	RV endo WT	1	18	2	2	2	2	2	2	2	2	2
	RV endo HET	1	18	2	2	2	2	2	2	2	2	2
	RV epi WT	1	18	2	2	2	2	2	2	2	2	2
	RV epi HET	1	18	2	2	2	2	2	2	2	2	2
	RVOT WT	1	18	2	2	2	2	2	2	2	2	2
	RVOT HET	1	18	2	2	2	2	2	2	2	2	2
adult ventricular cardiomyopathies	LV Sham MI	2	18	2	2	2	2	2	2	2	2	2
	3dpi BZ	6	36	4	4	4	4	4	4	4	4	4
	3dpi IZ	2	9	1	1	1	1	1	1	1	1	1
	7dpi IZ	2	9	1	1	1	1	1	1	1	1	1
	14dpi BZ	8	63	7	7	7	7	7	7	7	7	7
	14dpi IZ	2	8	1	1	1	0	1	1	1	1	1
	LV Sham TAC	2	18	2	2	2	2	2	2	2	2	2
	LV TAC	3	54	6	6	6	6	6	6	6	6	6
adult atria	Atria	2	13	2	0	1	2	1	2	2	2	1
	Adult LA	2	16	2	2	2	1	1	2	2	2	2
	Adult RA	2	19	3	2	2	2	1	2	2	2	3
cell lines	HL1	2	18	2	2	2	2	2	2	2	2	2
in vitro experiments	HL1 Bmp	2	18	2	2	2	2	2	2	2	2	2
	HL1 Tsa	1	9	1	1	1	1	1	1	1	1	1

Abbreviations

Heart parts: WH = whole heart / LA = left atrium / RA = right atrium / LV = left ventricle / RV = right ventricle / epi = epicardium and external myocardium / endo = endocardium and internal myocardium / RVOT = right ventricular outflow tract

Stages: e = embryonic day / p = postnatal / dpi = days post-infarction

Treatments: Sham = sham / MI = myocardial infarction / BZ = border zone / IZ = infarct zone / TAC = Transverse Aortic Constriction / Bmp = bone morphogenetic protein / TSA = Trichostatin A

Supplementary Table 3.

PCR efficiency of the different genes that were considered as candidate reference genes.
An efficiency of 2 means a doubling per cycle or a 100% efficient PCR. N indicates the number amplification curves used to determine the PCR efficiency. SEM = standard error of the mean; RSE = relative SE calcualted as SEM/efficiency.

Reference gene candidates

	PCR Efficiency	SEM	RSE	N
Eef1e1	1.887	0.0045	0.0024	99
H2afz	1.879	0.0043	0.0023	85
Hprt1	1.906	0.0038	0.0020	99
Pgk1	1.891	0.0048	0.0026	97
Ppia	1.905	0.0044	0.0023	99
Polr2a	1.887	0.0045	0.0024	85
Rpl32	1.904	0.0042	0.0022	100
Rpl4	1.907	0.0043	0.0023	100
Tbp	1.872	0.0034	0.0018	90

Validation experiment

	PCR Efficiency	SEM	RSE	N
Bact	1.832	0.0050	0.0027	26
GAPDH	1.878	0.0056	0.0030	26
Fstl1	1.839	0.0051	0.0028	49
Fstl5	1.898	0.0050	0.0026	25

Legends

Supplemental Figure1

Electrophoresis gel showing the products of a qPCR run with different primer sets and input murine gDNA, cDNA, -RT and NTC without DNAse treatment. The expected bands are shown in the cDNA lanes, the pseudogenes are clearly visible in the gDNA lanes except for Eef1e1, Tbp and Polr2a. The -RT lanes show in most cases a band on the position of the band in the cDNA lanes, which shows the necessity of a DNAse treatment. The NTC lanes show no products. In between every primer pair there is a pBluescript Msp1 marker for reference of the expected product size. The primers for Actb were discarded because of this analysis. Abbreviations: gDNA = genomic DNA; cDNA = copy DNA; -RT = cDNA synthesis reaction without reverse transcriptase; NTC = no tissue control (Water)

Supplemental Figure 2.

M and V graphs. M-values (indicating gene stability) and V-values (indicating the required number of genes) are given for each of the experimental designs. See Table 3 in the text for the tissue types that are included in the experimental design. Per condition, the candidate reference genes are ranked on stability (M-value). A V-value below 0.15 (red line) indicates a sufficiently stable subset of reference genes.

Supplemental. Figure 3.

Effects of an incorrect normalization strategy on observed Fstl1 expression. (A) Expression in whole heart during development (embryonic day 10.5 and 14.5, postnatal day 7 and adult mouse) using Eef1e1 and Rpl4 as reference genes for normalization (compare to Figure 1A). (B) Expression in left ventricle in normal condition or at different days after myocardial infarction (MI) using Ppia and Rpl32 as reference genes for normalization (compare to figure 1C). The normalized Follistatin-like 1 expression differs significantly between groups (one-way ANOVA). The insets show the result of the subsequent multiple comparison between groups. Grey boxes indicate subsets of groups with absence of evidence for differential expression (p-value < 0.05) according the Student-Newman-Keuls test.

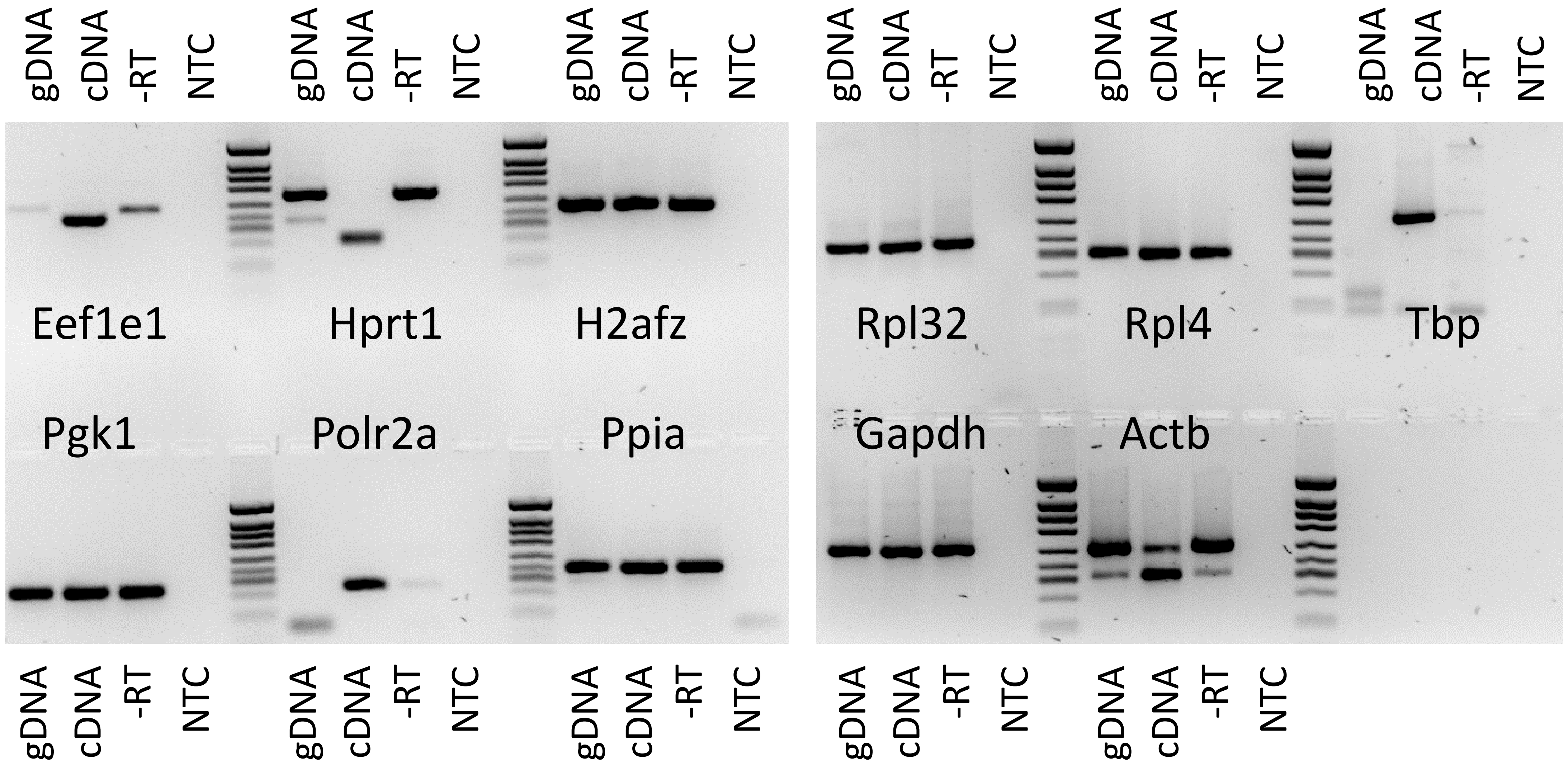
gDNA cDNA -RT NTC
gDNA cDNA -RT NTC
gDNA cDNA -RT NTC
gDNA cDNA -RT NTC
gDNA cDNA -RT NTC
gDNA cDNA -RT NTC
Eef1e1
Hprt1
H2afz
Rpl32
Rpl4
Tbp
Pgk1
Polr2a
Ppia
Gapdh
Actb
gDNA cDNA -RT NTC
gDNA cDNA -RT NTC
gDNA cDNA -RT NTC
gDNA cDNA -RT NTC
gDNA cDNA -RT NTC

Supplemental Figure 2

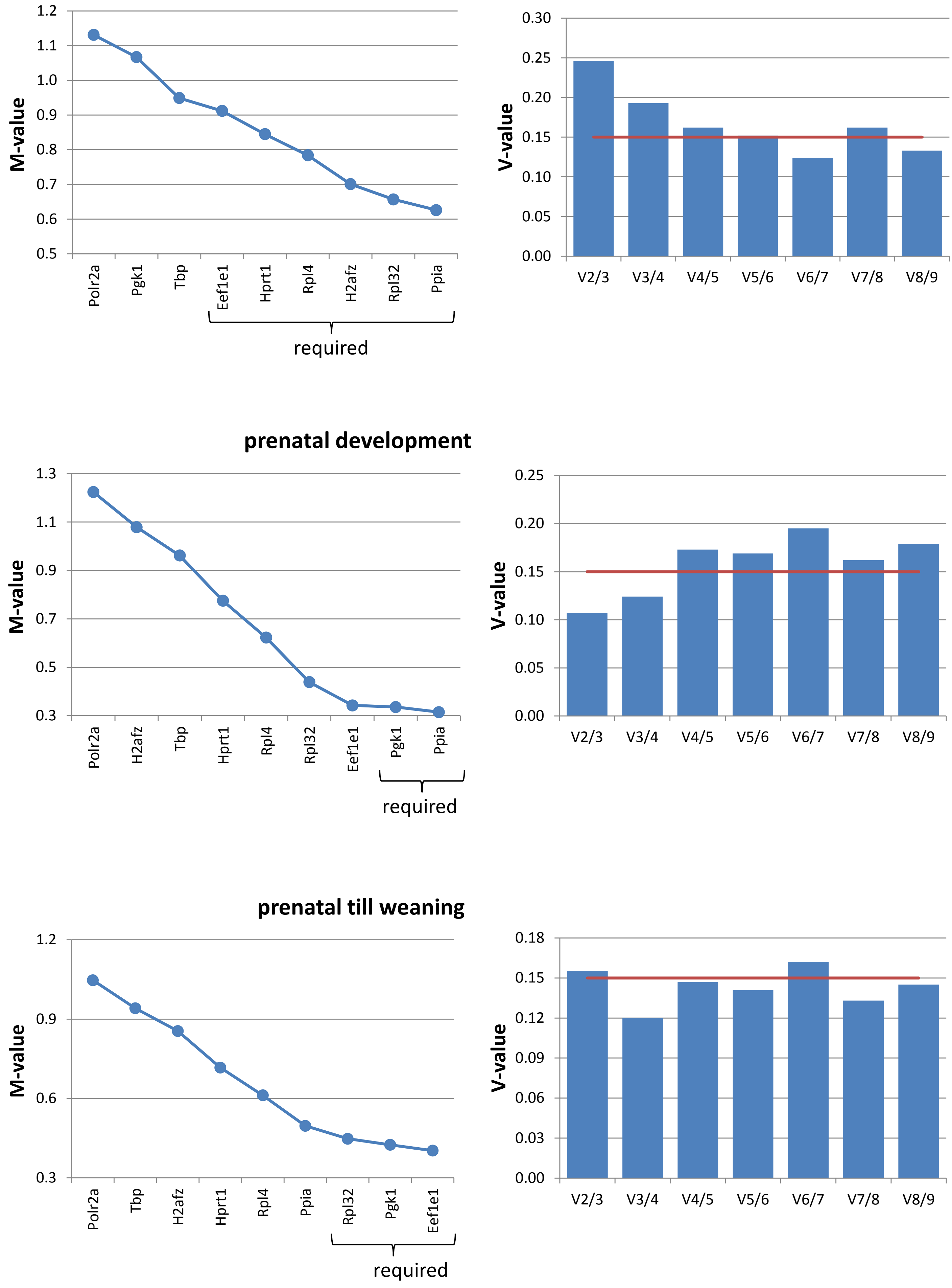

Supplemental Figure 2 (cont'd)

prenatal till adult

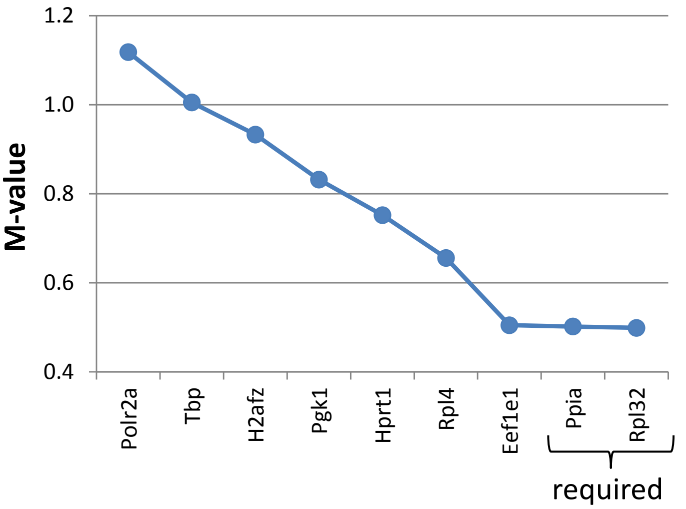

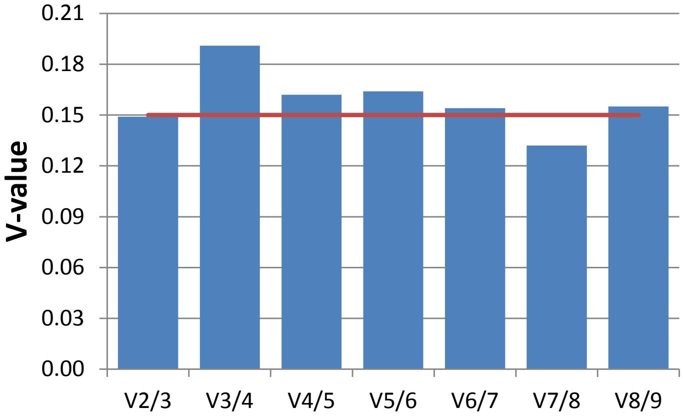

weaning till adult

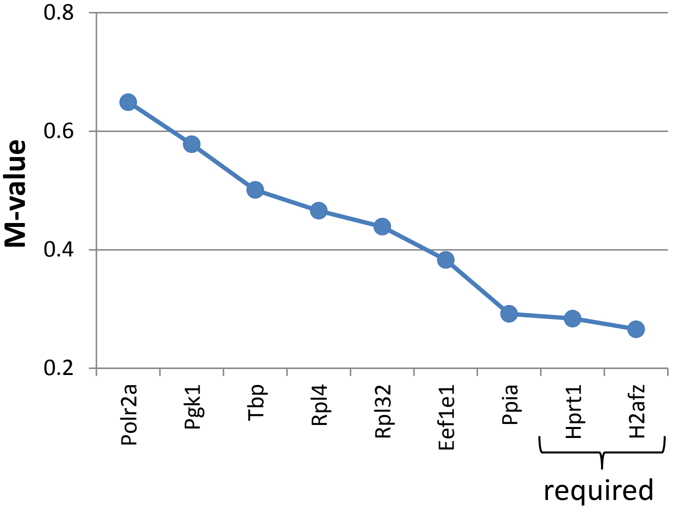

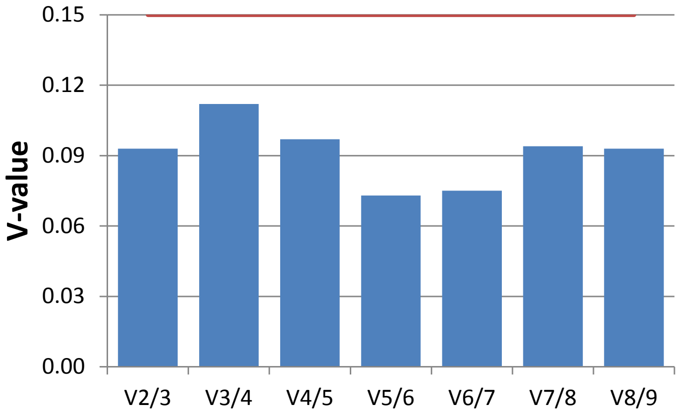

adult

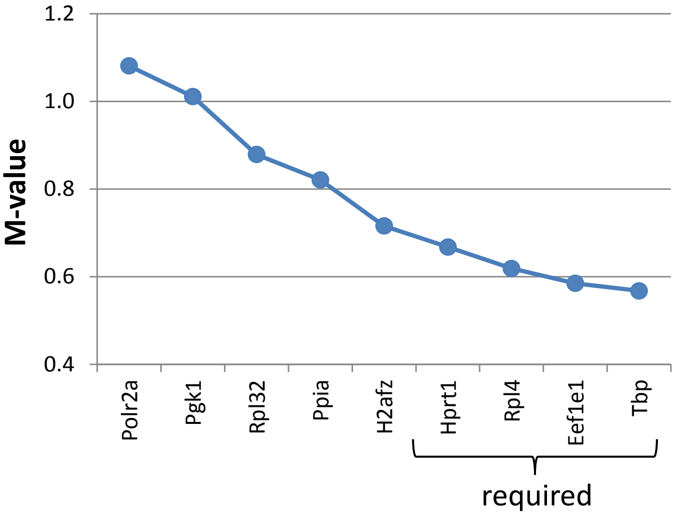

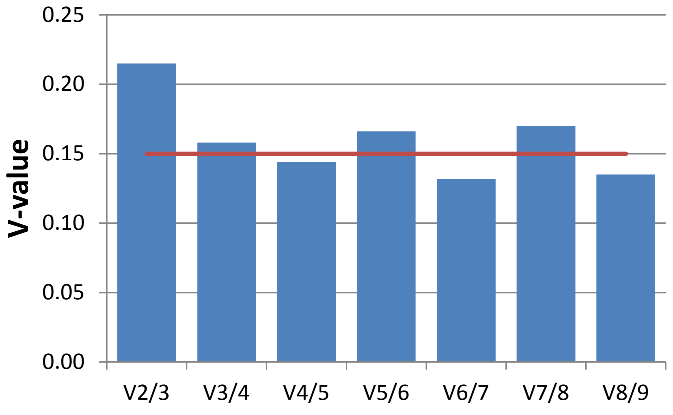

Supplemental Figure 2 (cont'd)

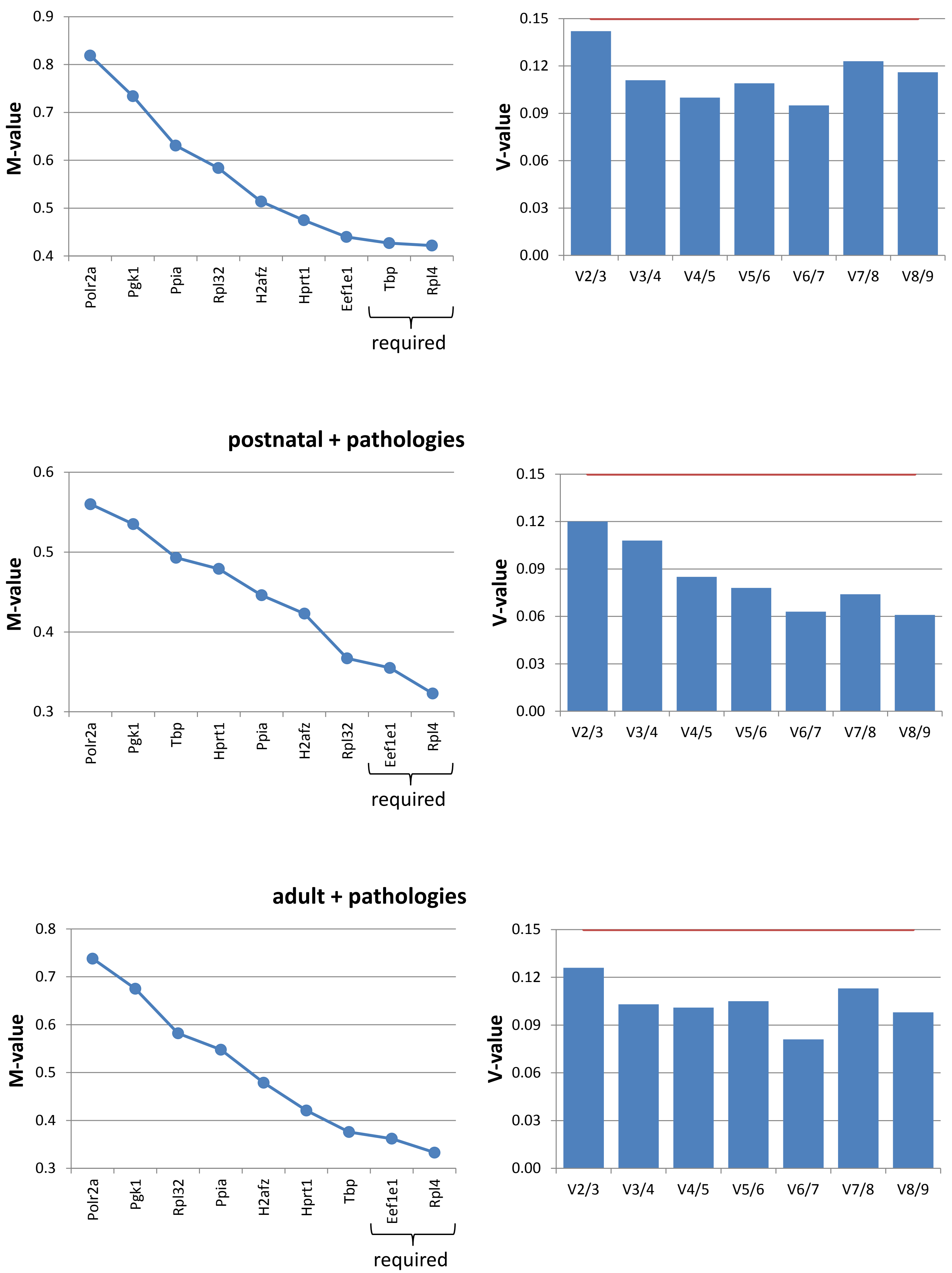

Supplemental Figure 2 (cont'd)

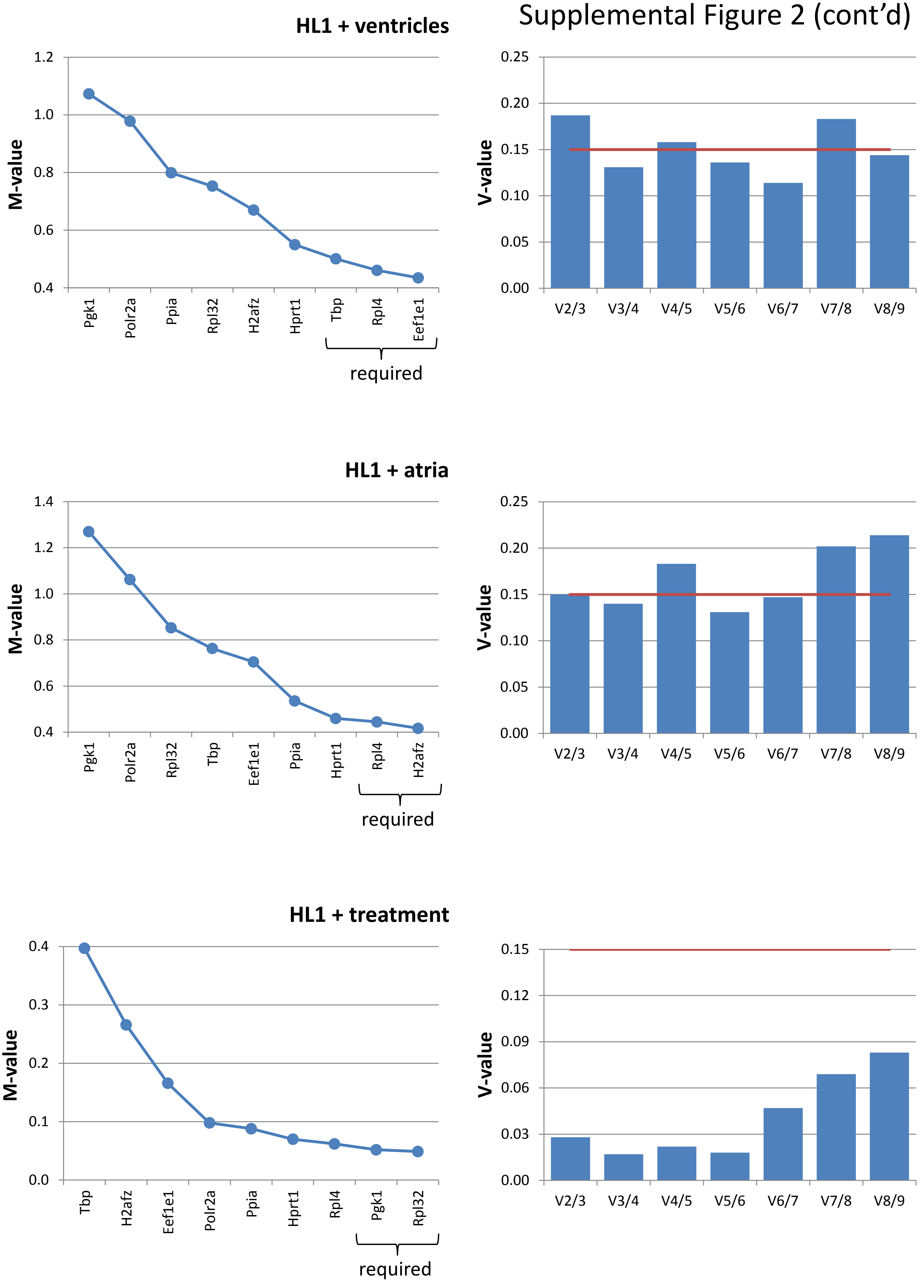

Supplemental Figure 3.
Observed Fstl1 expression after normalizations with the wrong set of reference genes

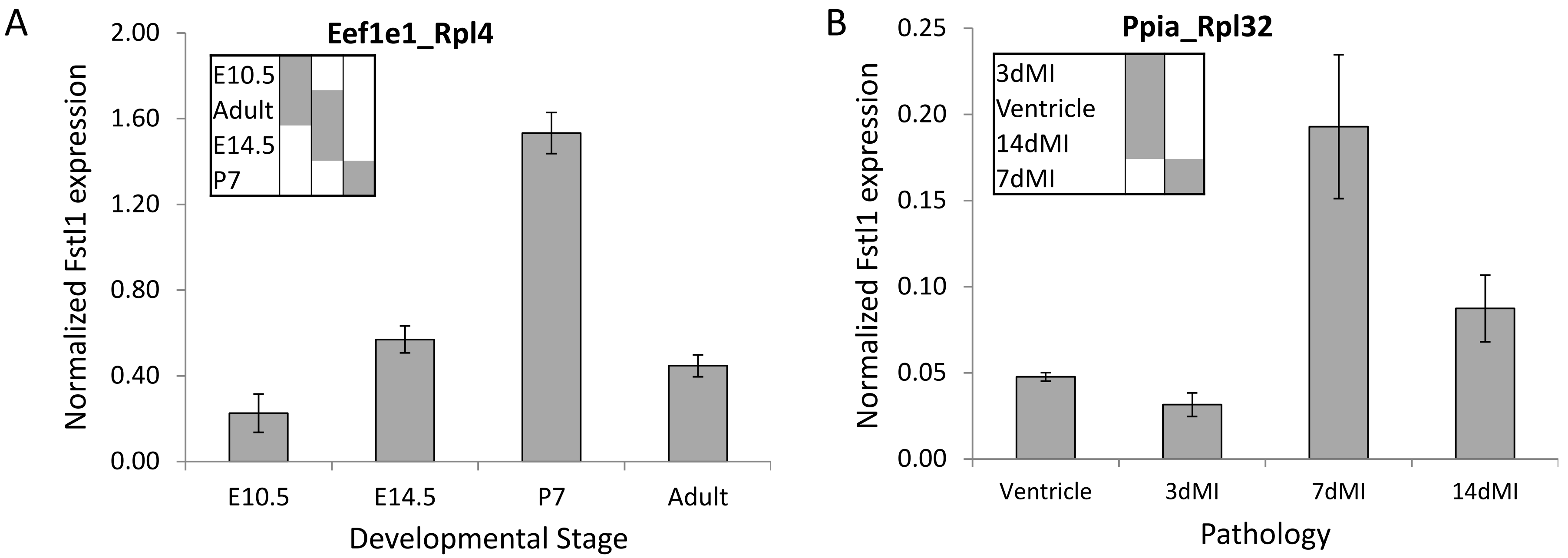

Supplement: Supplementary file 1 — Supplementary Material [file 41598_2017_43_MOESM1_ESM.pdf]
